# Orthostatic intolerance with tachycardia (postural tachycardia syndrome) and without (hypocapnic cerebral hypoperfusion) represent a spectrum of the same disorder

**DOI:** 10.3389/fneur.2024.1476918

**Published:** 2024-10-30

**Authors:** Peter Novak, David M. Systrom, Alexandra Witte, Sadie P. Marciano

**Affiliations:** ^1^Department of Neurology, Brigham and Women’s Hospital, Boston, MA, United States; ^2^Harvard University, Boston, MA, United States; ^3^Department of Medicine, Pulmonary and Critical Care, Brigham and Women’s Hospital, Boston, MA, United States

**Keywords:** HYCH, POTS, QASAT, dysautonomia, orthostatic intolerance

## Abstract

**Background:**

Spectrum of chronic orthostatic intolerance without orthostatic hypotension includes postural tachycardia syndrome (POTS), with orthostatic tachycardia and hypocapnic cerebral hypoperfusion (HYCH), without orthostatic tachycardia. This study compared autonomic, cerebrovascular, and neuropathic features of POTS and HYCH.

**Methods:**

This retrospective study evaluated patients with orthostatic intolerance referred for autonomic testing. Analyzed data included surveys (Survey of Autonomic Symptoms, Compass-31, Neuropathy Total Symptom Score-6, Central Sensitization Inventory) and autonomic tests (Valsalva maneuver, deep breathing, sudomotor and tilt tests), cerebrovascular (cerebral blood flow velocity (CBFv) monitoring in the middle cerebral artery), respiratory (capnography), neuropathic (skin biopsies for assessment of small fiber neuropathy) and invasive cardiopulmonary exercise testing (iCPET).

**Results:**

A total of 127 HYCH, 125 POTS, and 42 healthy controls were analyzed. Compared HYCH to POTS patients, there was no difference in the duration of symptoms, the prevalence of younger women, comorbidities, sensory and autonomic complaints, central sensitization syndrome, supine/standing norepinephrine levels, inflammatory markers and medical therapy except for gastrointestinal medication. Autonomic testing showed widespread but similar abnormalities in POTS and HYCH that included: reduced orthostatic CBFv and end-tidal CO_2,_ preload failure (assessed in 16/19 POTS/HYCH), mild autonomic failure, and frequent small fiber neuropathy.

**Conclusion:**

HYCH and POTS are syndromes of orthostatic intolerance with cerebral hypoperfusion associated with reduced orthostatic cerebral blood flow, hypocapnia, mild autonomic failure and small fiber neuropathy of a similar degree and distribution; except for tachycardia in POTS. Similarities in peripheral domain abnormalities that affect heart rate suggest that orthostatic tachycardia in POTS is driven by the central nervous system overcompensation of orthostatic challenge. These findings provide additional evidence that HYCH and POTS represent a spectrum of the same disorder. Reduced orthostatic cerebral blood flow is a key unifying feature of HYCH and POTS.

## 1 Introduction

Orthostatic intolerance syndromes without orthostatic hypotension have been increasingly recognized (1). These multi-system disorders are associated with various orthostatic and non-orthostatic symptoms. Characteristic orthostatic symptoms develop or are exacerbated upon standing up or even after prolonged sitting, and are relieved by lying down (1, 2). Typical orthostatic symptoms are indicative of cerebral hypoperfusion such as lightheadedness, dizziness, pre-syncope, cognitive problems, and fatigue; and those of cerebral hypoperfusion-triggered sympathetic excitation such as palpitations, anxiety, nausea, chest pain, and headaches (3). Common orthostatic intolerance syndromes are postural tachycardia syndrome (POTS) (3) where orthostatic tachycardia is present, and hypocapnic cerebral hypoperfusion (HYCH) (4), where orthostatic tachycardia is absent. Both syndromes are associated with symptoms of cerebral hypoperfusion. Objective testing usually shows reduced orthostatic cerebral blood flow due to hypocapnia-induced cerebral arteriolar vasoconstriction (4). It is estimated that POTS affects more than 0.2% of Americans (5). The prevalence of HYCH is unknown but it can be even more common than POTS (4). While POTS has been subjected to many studies, the profile of HYCH is less understood. Limited data suggests that HYCH and POTS have similar clinical presentations except for absent tachycardia in HYCH implying that they may represent a continuum of the same disorder (4).

This study aimed to compare HYCH with POTS to test the hypothesis that these conditions represent a spectrum of the same disorder of orthostatic intolerance without orthostatic hypotension. We compared the autonomic, cerebrovascular, and neuropathic features between HYCH and POTS.

## 2 Methods

This retrospective, single-center study evaluated consecutive adult patients with history of orthostatic intolerance who were referred for autonomic testing between 2018 and 2023 at the Brigham and Women’s Faulkner Hospital Autonomic Laboratory, Boston. Patients were included in the study if autonomic testing was consistent with POTS or HYCH and met the inclusion/exclusion criteria. Clinical data were obtained from patients’ electronic records.

### 2.1 Standard protocol approvals, registrations, and patient consent

The study was approved by the Institutional Review Board of the Brigham and Women’s Hospital, Harvard Medical School, as a minimal-risk study, and the consent form signature was waived.

### 2.2 Clinical definitions and selection criteria

Orthostatic intolerance was defined as the presence or exacerbation of chronic (>6 months) symptoms of cerebral hypoperfusion during standing that are relieved by recumbency. POTS is generally defined as a combination of orthostatic intolerance and a sustained increment in heart rate ≥ 30 beats per minute for patients older than 19 years (≥40 bpm, for 12–19 years old) without orthostatic hypotension during the tilt test (6). HYCH was previously defined as a combination of orthostatic intolerance and reduced orthostatic cerebral blood flow velocity (CBFv) associated with orthostatic hypocapnia (end-tidal CO_2_ < 30 mmHg) (7) without orthostatic tachycardia and orthostatic hypotension (8). To maximize the homogeneity of our diagnostic groups, we used the following inclusion criteria for both POTS and HYCH: (1) History of orthostatic intolerance; (2) Age < 50. For POTS, an additional inclusion criterion was sustained heart rate increment ≥30 beats per minute without orthostatic hypotension. Since there are various definitions of “sustained” orthostatic tachycardia (9) we defined sustained orthostatic tachycardia as orthostatic tachycardia that is equal to four minutes or longer during the head-up tilt. For HYCH, in addition to criteria (1) and (2), the inclusion criteria were a sustained decrease in orthostatic CBFv and end-tidal CO_2_ for four minutes or longer during the head-up tilt. The goal was to exclude patients with short-lasting tachycardic, cerebrovascular, and respiratory abnormalities that can be due to anxiety, particularly at the tilt onset (10).

The HYCH and POTS subjects were compared to a healthy control group with similar age, gender, and body mass index from our Autonomic research database at the University of Massachusetts (11). All controls were asymptomatic and had normal responses to tilt in heart rate, blood pressure, CBFv, and respiratory variables.

### 2.3 Patient reported surveys

Patient-reported surveys for sensory and autonomic symptoms were filled out by study participants before autonomic testing. The Survey of Autonomic Symptoms was used to assess the frequency and severity of autonomic symptoms (12). Composite Autonomic Symptoms Score (Compass)-31 was used to assess severity of autonomic symptoms in HYCH and POTS (13). Sensory complaints were assessed by the self-reported Neuropathy Total Symptom Score-6 (NTSS-6) (14).

The pain for the last seven days was assessed using the 0–10 numerical rating scale (0 = no pain, 10 = worst imaginable pain), a part of the NIH Toolbox (15), was assessed during the autonomic testing. The scores ≤3 correspond to mild, scores 4–6 to moderate, and scores ≥7 to severe pain (16). All subjects were observed for the presence of orthostatic symptoms during the head-up tilt test.

The presence of central sensitization was assesses using the central sensitization inventory, a validated instrument used for screening of central sensitization (17). A score equal to 40 is recommended as a cutoff for the detection of central sensitization syndrome.

### 2.4 Autonomic testing

All testing was performed following established standards and previously described in detail (8). Medications affecting the cardiovascular and autonomic nervous system were discontinued for five half-lives or longer before the testing. Cardiovascular reflex tests included deep breathing (evaluating cardiovagal parasympathetic function), the Valsalva maneuver (evaluating sympathetic adrenergic function using blood pressure responses and cardiovagal parasympathetic function using the heart rate responses), and the tilt test (evaluating both sympathetic and parasympathetic function). Deep breathing test was performed at the duration of inhalation and exhalation each equal to ten seconds for six respiratory cycles. Parasympathetic cardiovagal index was obtained as the average difference between expiratory and inspiratory heart rate. Valsalva maneuver was performed as a forced expiration at the expiratory pressure 40 mmHg for 15 s. The difference between baseline and end of the phase 2 in mean blood pressure was used as a sympathetic adrenergic index (11). Autonomic, respiratory and cerebral blood flow measurements from the tilt were previously described in details (8) and were calculated using the qpack package (18).

Patients were tilted at 70 degrees for 10 min following 10 min of supine rest. Recorded signals included electrocardiogram, blood pressure, end-tidal CO_2_, and CBFv in the middle cerebral artery using Transcranial Doppler. Blood pressure was obtained intermittently every minute through brachial sphygmomanometry using an automated monitor Welch Allyn CVSM 6400 Monitor (Skaneateles Falls, NY) and continuously using the photoplethysmographic signal volume-clamped in the finger by servo control (Human NIBP Nano Interface MLA382, ADInstruments Inc., Colorado Springs, CO, USA and Human NIBP Nano Wrist Unit FMS910804, Finapress Medical Systems, Amsterdam, Netherlands). End-tidal CO_2_ was obtained using the Nonin Respsense Capnograph (Nonin Medical Inc. Plymouth, MN) via a nasal cannula. A pulse oximeter (part of the Welch Allyn monitor) was used to monitor the oxygen saturation throughout the testing. The temporal acoustic window with a 2 MHz probe was used for the acquisition of CBFv using a MultiDop T (Multigon, New York, NY). The right middle cerebral artery has been insonated at a depth between 45 and 65 mm. The transducer has been attached to the head using a head frame with a three-dimensional positioner. The depth and angle of insonation were kept constant throughout the head-up tilt test. Signals were recorded using the PowerLab 16/35 data acquisition system with LabChart 8 software (ADInstruments Inc., Colorado Springs, CO, USA) and sampled at 400 Hz.

The normative threshold for the lower limit of the supine CBFv is (women/men) 82.2–0.45 (cm/s)*age (years)/72.09–0.38 (cm/s)*age (years) (8). The normal threshold for the orthostatic reduction in CBFv in percent is defined (in percent) using the following equation: normative value (%) = 90.36%–0.443 * minute of the tilt. For example, at the 3rd minute of the head-up tilt, the threshold for the normal value will be 90.36–0.443*3 = 89% of the supine baseline, which is equal to a drop of CBFv by 11%. Electrochemical skin conductance (ESC) was used to measure the sudomotor function (8).

### 2.5 Skin biopsies

Epidermal nerve fiber density (ENFD) and sweat gland nerve fiber density (SGNFD) were obtained using established standards (19, 20). Skin samples were taken from the proximal thigh 20 cm distal to the iliac spine and the calf 10 cm above the lateral malleolus using a 3-mm circular punch tool. Skin samples were immunoperoxidase-stained for the axonal marker protein gene product 9.5 (PGP 9.5). Skin processing and fiber counting was done at Therapath (New York, NY).

### 2.6 Invasive cardiopulmonary exercise testing (iCPET)

Medical records were also searched for an iCPET (21). iCPET is an invasive test indicated for evaluation of unexplained fatigue. During iCPET ventilation, an incremental cycling protocol measures pulmonary and systemic gas exchange and pressures. The iCPET calculates cardiac output and measures biventricular filling pressures, variables that are characteristically low in POTS (22). The iCPET was performed in a sitting position using a cycle ergometer as described in detail (23).

### 2.7 Grading of autonomic testing and skin biopsies

Test results were graded using the Quantitative Scale for Grading of Cardiovascular Autonomic Reflex Tests and Small Fibers from Skin Biopsies (QASAT) (8, 18). QASAT is an objective instrument for grading the intensity of dysautonomia, small fiber neuropathy, and cerebral blood flow abnormalities. Each domain (heart rate, blood pressure, cerebral blood flow, end-tidal CO_2_) is analyzed separately, where a score equal to 0 is normal, and above 0 is abnormal. QASAT gradings are defined (18):

QASAT_af=_QASAT_cardiovagal_ + QASAT_adrenergic_ + QASAT_sudomotor.;_ where QASAT_af_ is the autonomic failure score. QASAT_cardiovagal_ is the cardiovagal failure score and is obtained from heart rate responses in deep breathing test; the QASAT_adrenergic_ is the adrenergic failure score obtained as the summation of blood pressure responses to Valsalva maneuver and to the head-up tilt scores; the QASAT_sudomotor_ is sudomotor failure score obtained from ESC. The range of QASAT_af_ is 0–22. QASAT_af_ is defined as normal (0), mildly abnormal (1–3), moderately abnormal (4–12), and severely abnormal (12–22). The additional QASAT ranges were defined as follows: cardiovagal failure: none (0), abnormality: mild (1), moderate (2) and severe (3); adrenergic failure – Valsalva maneuver: none (0), abnormality: mild (1), moderate (2) and severe (3); adrenergic failure – orthostatic hypotension: none (0), abnormality: mild (1), moderate (2–5) and severe (6–10); orthostatic tachycardia: none (0), abnormality: mild (1–2), moderate (3–5) and severe (6–10); sudomotor failure – ESC: none (0), abnormality: mild (1–2), moderate (3–4) and severe (5–6); sudomotor failure – QSART: none (0), abnormality: mild (1–2), moderate (3–6) and severe (7–8); Epidermal nerve fiber density (ENFD): normal (0), abnormality: mild (1–2), moderate (3–6) and severe (7–8); Sweat gland nerve fiber density (SGNFD): normal (0), abnormality: mild (1–2), moderate (3–6) and severe (7–8); reduced orthostatic end-tidal CO_2_: normal/none (0), abnormality: mild (1–2), moderate (3–5) and severe (6–10); reduced orthostatic CBFv: normal/none (0), abnormality: mild (1–2), moderate (3–5) and severe (6–10). Details of calculations and grading of the testing were published previously (18).

### 2.8 Statistical analysis

Group comparisons used ANOVA for the continuous variables and chi-squared test for the categorical variables. If the group comparisons were significant, pairwise comparisons between HYCH and POTS were done using Tukey’s Honestly Significant Difference test for continuous variables and by Fisher Exact test adjusted by Holm method for categorical variables. The effect of head-up tilt on CBFv was assessed using the linear mixed-effects models with a repeated-measures design. The predictor variables were the end-tidal CO_2_, heart rate, blood pressure, diagnosis, and position (supine vs. upright) during the head-up tilt, gender and age. The relationship between lightheadedness during head-up tilt (absent versus present) and QASAT domains was evaluated using the binary logistic regression model. Missing data were ignored. A *p*-value <0.05 adjusted for multiple comparisons was considered significant. The R software (https://www.r-project.org) was used for statistical analyses.

## 3 Results

From a total 3,327 patients who underwent autonomic testing for evaluation of orthostatic intolerance, 127 HYCH and 125 POTS patients satisfied the inclusion criteria and were included in this study and they were compared to 42 healthy controls (Table 1). HYCH and POTS patients had similar age and gender, duration of symptoms, comorbidities, and medical therapy except gastrointestinal symptoms treatment (stool softeners, pro-motility agents, antiemetics) was more frequently used in HYCH (*p* = 0.001). The use of medications for orthostatic tachycardia (*β* adrenergic blockers, calcium channel blockers, ivabradine) and of pressor medication (pyridostigmine, fludrocortisone, proamatine, droxydopa) was similar in both groups. Laboratory evaluations were similar between both HYCH and POTS.

**Table 1:** Demographic and baseline characteristics.

Variable	Control (*n* = 42)	HYCH (*n* = 127)	POTS (*n* = 125)	*p* value	
				Overall^a^	HYCH-POTS^b^
Age, years	33.14 (8.12)	33.17 (8.03)	31.54 (7.73)	0.226	0.236
Gender, female %	85.7	86.6	92.0	0.104	0.665
Race					
African American, %	0.0	0.8	0.0		
Asian, %	0.0	0.8	0.0		
Multiracial, %	0.0	0.0	0.8		
White, %	100.0	98.4	99.2		
BMI,m^2^/kg	25.21 (3.83)	25.13 (4.90)	23.98 (5.47)	0.143	0.165
Symptoms duration,years	0.00 (0.00)	7.17 (5.86)	6.31 (4.95)	<0.001	0.365
Comorbid conditions					
Hypermobile Ehlers-Danlos syndrome, %	0.0	18.9	15.2	0.104	0.504
Myalgic encephalitis/Chronic fatigue syndrome, %	0.0	14.2	15.2	0.104	0.86
Diabetes mellitus, %	0.0	0.8	0.8	0.104	1
Lyme disease, chronic, %	0.0	4.7	6.4	0.104	0.676
Mast cell activation syndrome, %	0.0	12.6	9.6	0.104	0.549
Hereditary alpha tryptasemia, %	0.0	2.4	2.4	0.104	1
Depression, %	0.0	63.2	66.7	0.104	0.825
Fibromyalgia, %	0.0	15.8	14.5	0.104	1
Irritable bowel syndrome, %	0.0	47.4	27.3	0.104	0.103
Anxiety, %	0.0	68.4	76.4	0.104	0.477
Headaches, %	0.0	63.2	63.6	0.104	1
Medication					
Anti-histamine, %	0.0	47.2	41.6	0.104	0.378
Pain, %	0.0	42.5	37.6	0.104	0.443
Pressor, %	0.0	27.6	33.6	0.104	0.339
Psychiatric, %	0.0	51.2	48.8	0.104	0.801
Hypertension, %	0.0	6.3	3.2	0.104	1
Antitachycardic, %	0.0	15.0	22.4	0.104	0.147
Gastrointestinal, %	0.0	37.8	17.6	0.104	<0.001
Immmunomodulators, %	0.0	3.9	8.8	0.104	0.379
Laboratory evaluations					
C-reactive protein-high sensitivity, normal ≤3 mg/L	Na	2.81 (4.37)	1.43 (2.33)		0.0668
C-reactive protein-high sensitivity, % abnormal	Na	23.4	11.4		0.172
Interleukin 6, normal <7.1 pg./mL	Na	2.87 (0.66)	2.69 (0.61)		0.347
Interleukin 6, % abnormal	Na	4.5	4.3		1
Interleukin 1b, normal <0.1 pg./mL	Na	0.20 (0.21)	1.70 (5.96)		0.294
Interleukin 1b, % abnormal	Na	5.6	4.8		1
Tumor necrosis factor alpha, normal ≤2.8 pg./mL	Na	2.92 (4.33)	4.09 (6.86)		0.484
Tumor necrosis factor alpha, % abnormal	Na	9.5	23.1		0.269
Leptin, normal range = 3.3–18.3 ng/mL	Na	14.75 (10.68)	10.60 (10.83)		0.271
Leptin, % abnormal	Na	0.29 (0.47)	0.17 (0.38)		0.399
Tryptase, normal<11.5 ng/mL	Na	4.70 (2.99)	3.93 (2.35)		0.145
Tryptase, % abnormal	Na	7.8	2.0		0.363
Voltage gated potassium channel complex antibody, normal ≤0.02 nmol/L	Na	0.01 (0.09)	0.00 (0.00)		0.275
Voltage gated potassium channel complex antibody, % abnormal	Na	3.4	0.0		0.235
Calcium channel P/Q antibody, normal ≤0.02 nmol/L	Na	0.00 (0.00)	0.00 (0.01)		0.336
Calcium channel P/Q antibody, % abnormal	Na	0.0	1.6		1
Trisulfated heparin disaccharide antibody normal titer<10,000	Na	9647.06 (13431.94)	6127.27 (8499.09)		0.206
Trisulfated heparin disaccharide antibody, % abnormal	Na	38.2	36.4		1
Fibroblast growth factor receptor 3 antibody, normal titer<3,000	Na	1216.22 (2159.80)	2212.12 (4903.81)		0.267
Fibroblast growth factor receptor 3 antibody, % abnormal	Na	19.4	21.2		1
Neutrophil, normal range = 1.8–7.7 K/uL	Na	3.70 (1.34)	4.14 (1.71)		0.263
Neutrophil, % abnormal	Na	100.0	100.0		1
Lymphocyte, normal range = 1.0–4.8 K/uL	Na	1.81 (0.79)	2.01 (0.62)		0.261
Lymphocyte, % abnormal	Na	0.0	0.0		NA
Neutrophil/Lymphocyte ratio	Na	2.30 (0.83)	2.32 (1.23)		0.962
Platelet, normal range = 150–400 K/uL	Na	262.97 (69.51)	259.89 (55.06)		0.848
Platelet, % abnormal	Na	0.0	0.0		NA
Norepinephrine supine, normal 70–750 pg./mL,	Na	462.62 (267.19)	444.71 (203.74)		0.78
Norepinephrine supine, % abnormal	Na	9.5	5.9		0.632
Norepinephrine standing, normal 200–1700 pg./mL	Na	566.10 (279.90)	638.59 (241.10)		0.32
Norepinephrine standing, % abnormal	Na	14.3	15.6		1
Cortisol, normal range = 6.0–18.4 ug/dL	Na	10.83 (8.01)	13.25 (7.98)		0.682
Cortisol, % abnormal	Na	33.3	40.0		1
ACTH, normal range = 7.2–63 pg./mL	Na	15.00 (2.83)	48.67 (51.87)		0.448
ACTH, % abnormal	Na	0.0	66.7		1
Myoglobin, normal ≤71 ng/mL	Na	28.46 (22.17)	23.88 (10.20)		0.452
Myoglobin, % abnormal	Na	6.2	0.0		1
Ferritin, normal range = 20–300 ug/L	Na	91.00 (100.77)	68.47 (79.87)		0.603
Ferritin, % abnormal	Na	0.0	30.0		0.228

Antitachycardic: adrenergic beta-blockers, calcium channel blockers, ivabradine. Data are mean ± sd, %prevalence of respective variable in percent. ^a^Calculated using ANOVA or chi-squared test as appropriate. ^b^Pairwise comparison calculated using Tukey HSD or Fisher Exact test as appropriate.

### 3.1 Symptoms

HYCH and POTS patients reported complaints in all autonomic and sensory domains (Tables 2–4) but there was no difference between HYCH and POTS in all comparisons. Comparing all groups (controls, HYCH and POTS), there was an overall difference in total scores and subscores in SAS and NTSS-6 (Tables 2, 4). Compass-31 scores were not available for controls. The prevalence of central sensitization syndrome was equal in both groups (HYCH = 87.9%, POTS = 87.6%, *p* = 1).

**Table 2:** Survey of autonomic symptoms scores.

Domain	Control (*n* = 42)	HYCH (*n* = 127)	POTS (*n* = 125)	*p* value	
				Overall^a^	HYCH-POTS^b^
Total score	0.05 (0.22)	26.31 (10.40)	25.10 (9.83)	<0.001	0.562
Orthostatic	0.02 (0.15)	3.76 (1.32)	3.98 (1.04)	<0.001	0.23
Sudomotor	0.02 (0.15)	7.64 (4.95)	6.83 (4.67)	<0.001	0.325
Vasomotor	0.00 (0.00)	6.10 (3.02)	5.93 (2.79)	<0.001	0.865
Gastrointestinal	0.00 (0.00)	7.46 (3.71)	7.22 (3.67)	<0.001	0.842
Urinary	0.00 (0.00)	1.24 (1.59)	0.94 (1.44)	<0.001	0.207

Data are mean ± sd, ^a^Calculated using ANOVA, ^b^Pairwise comparison calculated using Tukey HSD test.

**Table 3:** Compass-31 scores.

Domain	HYCH (*n* = 127)	POTS (*n* = 125)	*p*-value^a^
Total	46.32 (15.44)	47.86 (13.40)	0.576
Orthostatic	23.18 (8.64)	25.97 (8.25)	0.0879
Vasomotor	2.57 (1.62)	2.84 (1.39)	0.342
Secretomotor	5.07 (3.95)	4.71 (3.78)	0.631
Gastrointestinal	11.13 (4.06)	10.44 (4.24)	0.385
Urinary	1.68 (1.95)	1.54 (1.36)	0.668
Pupillomotor	2.68 (1.26)	2.47 (1.13)	0.343

Data are mean ± sd, ^a^Calculated using ANOVA.

**Table 4:** Neuropathy Total Symptom Score-6.

Domain	Control (*n* = 42)	HYCH (*n* = 127)	POTS (*n* = 125)	*p* value	
				Overall^a^	HYCH-POTS^b^
Total	0.00 (0.00)	10.69 (5.39)	9.92 (5.31)	<0.001	0.437
Aching frequency	0.00 (0.00)	2.39 (0.97)	2.26 (1.10)	<0.001	0.573
Aching intensity	0.00 (0.00)	1.83 (0.86)	1.79 (0.98)	<0.001	0.944
Allodynia frequency	0.00 (0.00)	1.26 (1.10)	1.06 (1.18)	<0.001	0.278
Allodynia intensity	0.00 (0.00)	1.31 (1.17)	1.13 (1.26)	<0.001	0.387
Burning frequency	0.00 (0.00)	1.48 (1.15)	1.31 (1.15)	<0.001	0.425
Burning intensity	0.00 (0.00)	1.22 (1.04)	1.14 (1.03)	<0.001	0.765
Lancinating frequency	0.00 (0.00)	1.53 (1.19)	1.48 (1.25)	<0.001	0.941
Lancinating intensity	0.00 (0.00)	1.47 (1.15)	1.41 (1.24)	<0.001	0.889
Prickling frequency	0.00 (0.00)	2.00 (1.07)	1.96 (1.06)	<0.001	0.944
Prickling intensity	0.00 (0.00)	1.65 (0.98)	1.52 (0.93)	<0.001	0.456
Numbness frequency	0.00 (0.00)	1.90 (1.23)	1.82 (1.14)	<0.001	0.855
Numbness intensity	0.00 (0.00)	1.63 (1.08)	1.44 (0.99)	<0.001	0.26
Pain, NRS	0.00 (0.00)	3.04 (2.61)	2.90 (2.64)	<0.001	0.898

Data are mean ± sd, ^a^Calculated using ANOVA, ^b^Pairwise comparison calculated using Tukey HSD test.

### 3.2 Autonomic testing

**Figure 1:**
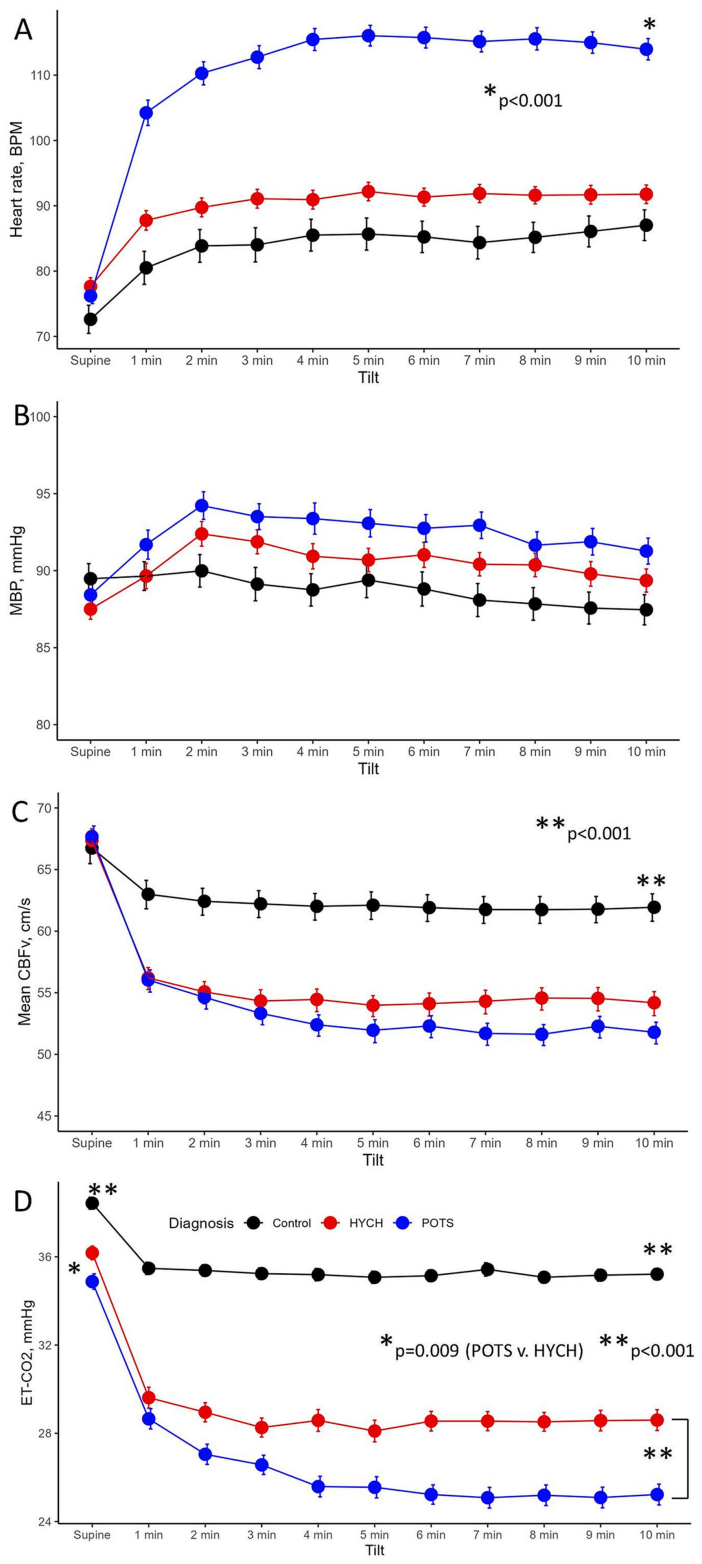
The head-up tilt profile showing hemodynamic variables at supine baseline and at every minute of the 10-min head-up tilt in the Control, HYCH-Hypocapnic Cerebral Hypoperfusion syndrome and POTS–Postural Tachycardia Syndrome; Data showing mean ± standard error. **(A)** Heart rate; **(B)** mean blood pressure; **(C)** mean CBFv in the middle cerebral artery; **(D)** end-tidal CO_2_. BPM, beats/min; MBP, mean blood pressure; CBFv, cerebral blood flow velocity; ET-CO2, end-tidal CO_2_; HYCH, hypocapnic cerebral hypoperfusion, POTS, postural tachycardia syndrome.

Comparing all groups, in the supine position there was no difference in all tested variables except for lower end-tidal CO_2_ in POTS (*p* = 0.0094, Table 5; Figure 1).

**Table 5:** Results of autonomic testing.

Variable	Control (*n* = 42)	HYCH (*n* = 127)	POTS (*n* = 125)	*p* value	
				Overall^a^	HYCH-POTS^b^
Heart rate supine, bpm	73.14 (14.95)	77.66 (14.83)	76.21 (13.26)	0.132	0.69
Heart rate orthostatic, bpm	87.02 (15.24)	91.76 (15.97)	113.96 (18.24)	<0.001	<0.001
Systolic BP supine, mmHg	118.60 (7.33)	114.39 (8.96)	115.66 (11.93)	0.067	0.578
Systolic BP orthostatic, mmHg	113.76 (9.42)	112.69 (10.84)	114.78 (12.38)	0.311	0.304
Mean BP supine, mmHg	89.48 (6.34)	87.50 (7.40)	88.42 (8.63)	0.328	0.617
Mean BP orthostatic, mmHg	87.89 (6.73)	89.33 (8.49)	91.27 (9.46)	0.032	0.186
Diastolic BP supine, mmHg	74.93 (6.68)	74.06 (7.23)	74.80 (7.50)	0.66	0.695
Diastolic BP orthostatic, mmHg	74.95 (6.09)	77.65 (7.69)	79.52 (8.56)	0.003	0.156
Systolic CBFv supine, cm/s	107.88 (11.57)	103.91 (16.32)	104.42 (15.51)	0.339	0.964
Systolic CBFv orthostatic, cm/s	98.58 (10.49)	85.34 (16.60)	81.34 (15.31)	<0.001	0.098
Mean CBFv supine, cm/s	66.74 (8.16)	67.36 (11.91)	67.68 (11.02)	0.893	0.973
Mean CBFv orthostatic, cm/s	61.94 (7.32)	54.19 (11.77)	51.79 (10.53)	<0.001	0.18
Diastolic CBFv supine, cm/s	46.17 (7.64)	49.09 (10.73)	49.34 (9.56)	0.177	0.977
Diastolic CBFv orthostatic, cm/s	43.62 (7.06)	38.61 (10.15)	37.02 (9.11)	<0.001	0.367
Mean CBFv corrected for CO_2_ orthostatic, cm/s	67.88 (7.97)	66.16 (14.17)	66.23 (12.43)	0.729	0.999
Maximal decline in orthostatic mean CBFv, cm/s	5.76 (2.03)	19.77 (6.68)	21.58 (8.76)	<0.001	0.12
Maximal decline in orthostatic mean CBFv, %	−8.57 (2.52)	−29.21 (7.87)	−31.53 (9.68)	<0.001	0.067
Respiratory frequency supine, breaths per minute	14.88 (1.25)	15.13 (5.70)	15.14 (5.21)	0.991	1
Respiratory frequency orthostatic, breaths per minute	16.50 (1.20)	16.59 (7.53)	15.59 (6.90)	0.533	0.508
End-tidal CO_2_ supine, mmHg	38.43 (1.74)	36.18 (3.47)	34.88 (3.92)	<0.001	0.009
End-tidal CO_2_ orthostatic, mmHg	35.21 (1.60)	28.60 (5.32)	25.22 (5.28)	<0.001	<0.001
Minimal orthostatic end-tidal CO_2_, mmHg	34.45 (1.67)	24.20 (4.67)	21.93 (5.13)	<0.001	<0.001
Maximal decline in orthostatic end-tidal CO_2_, mmHg	3.98 (0.90)	11.98 (3.86)	12.95 (4.35)	<0.001	0.112
Maximal decline in orthostatic end-tidal CO_2_, %	−10.33 (2.24)	−33.25 (10.39)	−37.31 (12.03)	<0.001	0.006
CVRi supine, mmHg/cm/s	1.37 (0.20)	1.34 (0.28)	1.34 (0.25)	0.886	1
CVRi orthostatic, mmHg/cm/s	1.11 (0.18)	1.34 (0.31)	1.43 (0.35)	<0.001	0.037
Cerebrovascular reactivity, %/mmHg	1.53 (0.69)	1.79 (0.79)	1.81 (0.89)	0.15	0.973
Deep breathing, heart rate	16.55 (6.67)	16.48 (7.87)	16.90 (7.78)	0.902	0.899
Valsalva ratio, beats per minute	1.64 (0.22)	1.69 (1.01)	1.76 (0.32)	0.538	0.677
Valsalva maneuver, end of phase 2 decline, mmHg	−8.12 (9.20)	5.49 (14.49)	4.35 (14.21)	<0.001	0.787
QASAT-CBFv, tilt response	0.00 (0.00)	7.63 (2.03)	8.27 (2.03)	<0.001	0.020
QASAT-ET-CO_2_, tilt response	0.00 (0.00)	7.29 (2.13)	8.06 (1.94)	<0.001	0.004
QASAT-Autonomic failure	0.00 (0.00)	2.69 (1.92)	2.30 (2.07)	<0.001	0.206
QASAT-Cardiovagal	0.00 (0.00)	0.31 (0.63)	0.30 (0.64)	0.007	0.988
QASAT-Adrenergic	0.00 (0.00)	1.06 (0.95)	1.03 (0.95)	<0.001	0.976
QASAT-Orthostatic hypotension	0.00 (0.00)	0.00 (0.00)	0.00 (0.00)	NA	NA
QASAT-Orthostatic tachycardia	0.00 (0.00)	0.00 (0.00)	7.36 (2.01)	<0.001	<0.001
QASAT-Sudomotor	0.00 (0.00)	1.32 (1.48)	0.97 (1.49)	<0.001	0.103
QASAT-ENFD	0.00 (0.00)	1.16 (1.78)	1.18 (2.01)	<0.001	0.992
QASAT-SGNFD	0.00 (0.00)	0.83 (1.55)	0.87 (1.82)	0.005	0.999

BP, blood pressure; CBFv, cerebral blood flow velocity; CVRi, cerebrovascular resistance index; QASAT, Quantitative Scale for Grading of Cardiovascular Autonomic Reflex Tests and Small Fibers from Skin Biopsies; Data are mean ± sd; % = results in percent. ^a^Calculated using ANOVA or chi-squared test as appropriate, ^b^Pairwise comparison calculated using Tukey HSD test or Fisher Exact test as appropriate.

#### 3.2.1 Head-up tilt test

Orthostatic heart rate was the highest in POTS (*p* < 0.001, Figures 1A–D), as expected. Orthostatic CBFv was reduced in HYCH and POTS compared to controls (*p* < 0.001), and was lower in POTS compared to HYCH (*p* = 0.048). Orthostatic end-tidal CO_2_ was reduced in HYCH and POTS compared to controls (*p* < 0.001) and was lower in POTS compared to HYCH (Figure 1D, *p* < 0.001). POTS has a higher cerebrovascular resistance (*p* = 0.019). There was no difference in blood pressure. In all subjects, the oxygen saturation was within normal limits during the tilt test (range 96–99%).

The linear mixed model predicted CBFv (conditional R^2^ = 0.68, marginal R^2^ = 0.44) with a significant effect of end-tidal CO_2_ (*p* < 0.001), heart rate (*p* < 0.001), mean blood pressure (*p* = 0.025), diagnosis (*p* < 0.001) and head-up tilt (*p* < 0.001). The effect of age and gender was not significant (*p* = 0.06–0.758). The effect of individual predictors was as follows: end-tidal CO_2_ 38.2%, heart rate 25.5%, diagnosis 19.3% and mean blood pressure 1%.

#### 3.2.2 QASAT grading

Overall comparisons showed abnormal QASAT scores (>0) in all domains except for the orthostatic hypotension domain (score = 0) since orthostatic hypotension was absent in all subjects (Table 5). Controls had QASAT scores equal to zero for all domains.

**Figure 2:**
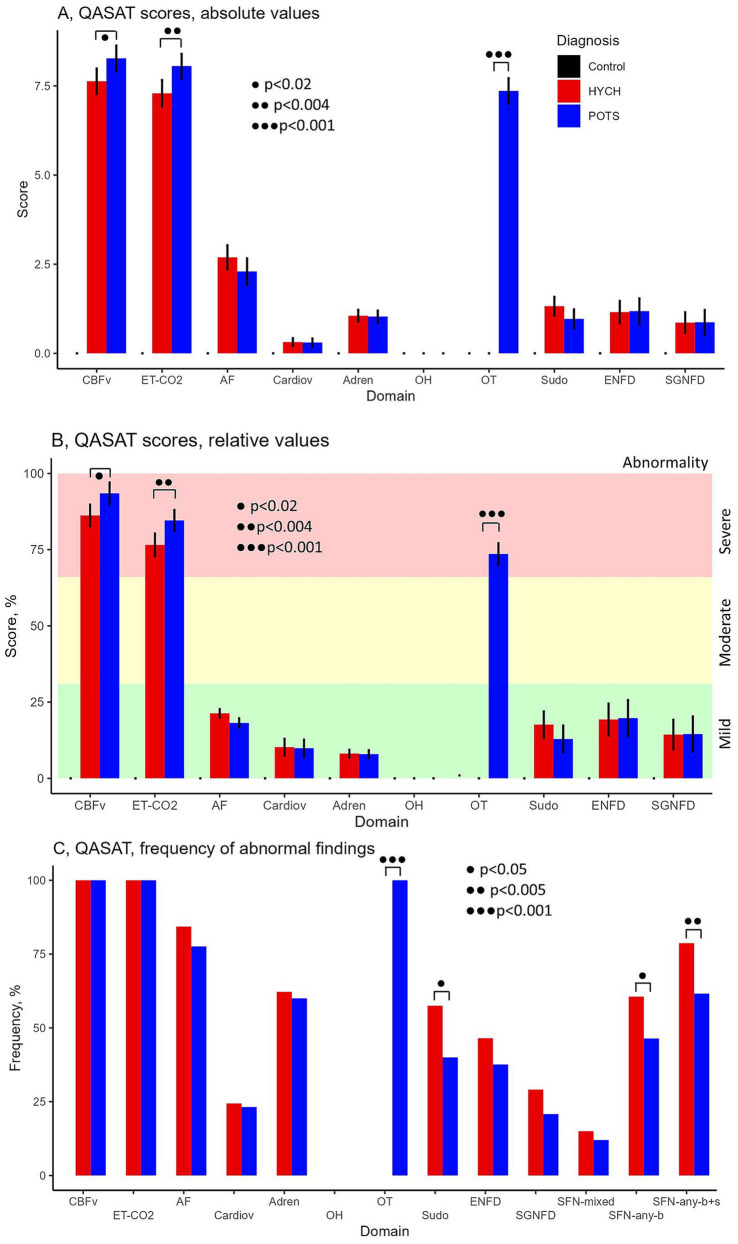
QASAT score results. **(A)** Absolute scores, mean ± sd. **(B)** Relative scores in percent, mean (%) ± sd. Each domain was normalized to the range 0–100% for easier comparisons. **(C)** Percentage of patients in which the QASAT score was abnormal (> 0). CBFv, cerebral blood flow velocity; ET-CO_2_, end-tidal; AF, autonomic failure, Cardiov, cardiovagal; Adren, adrenergic; OH, orthostatic hypotension; OT, orthostatic tachycardia; Sudo, sudomotor; ENFD, epidermal nerve fiber density, SGNFD, sweat gland nerve fiber density; SFN, small fiber neuropathy; SFN, any-b, SFN using morphological criteria (ENFD and SGNFD); SFN, any-b + s, using combined morphological and functional/sudomotor criteria. HYCH, hypocapnic cerebral hypoperfusion, POTS, postural tachycardia syndrome.

HYCH and POTS had similar degree of mild autonomic failure (QASAT_af_ score range 1–3, *p* = 0.116). HYCH and POTS also had similar cardiovagal (*p* = 0.891), adrenergic (*p* = 0.847), and sudomotor scores (*p* = 0.059), which were consistent with mild abnormalities. In contrast, orthostatic CBFv and end-tidal CO_2_ scores were consistent with severe abnormalities. Comparing HYCH to POTS, HYCH patients had lower orthostatic CBFv scores (*p* = 0.013) and lower orthostatic end-tidal CO_2_ scores (*p* = 0.003). ENFD and SGNFD scores were abnormal (>0) and similar compared to HYCH and POTS. The frequency of abnormalities on autonomic testing was similar in both disorders (Table 6; Figures 2A–C) except for the presence of orthostatic tachycardia in POTS. HYCH had borderline higher frequency small fiber neuropathy (*p* = 0.07).

**Table 6:** Frequency of abnormal findings from autonomic testing.

Variable	Control (*n* = 42)	HYCH (*n* = 127)	POTS (*n* = 125)	Overall P-value^a^	HYCH-POTS P values^b^
Orthostatic lightheadedness/dizziness, %	0.0	96.1	96.8	<0.001	1
Orthostatic dyspnea, %	0.0	74.8	73.6	<0.001	0.886
QASAT-CBFv, reduced during the tilt, %	0.0	100.0	100.0	<0.001	1
QASAT-End-tidal CO_2_, reduced during the tilt, %	0.0	100.0	100.0	<0.001	1
QASAT-Autonomic failure, %	0.0	84.3	77.6	<0.001	0.201
QASAT-Cardiovagal, %	0.0	24.4	23.2	0.002	0.883
QASAT-Adrenergic, %	0.0	62.2	60.0	<0.001	0.796
QASAT-Orthostatic tachycardia, %	0.0	0.0	100.0	<0.001	<0.001
QASAT-Sudomotor, %	0.0	57.5	40.0	<0.001	0.006
QASAT-ENFD, %	0.0	46.5	37.6	<0.001	0.163
QASAT-SGNFD, %	0.0	31.3	23.9	0.001	0.183
SFN, mixed, %	0.0	15.1	12.7	0.031	0.579
SFN, any from biopsy, %	0.0	62.6	50.0	<0.001	0.053
SFN, any, %	0.0	79.4	63.1	<0.001	0.005

QASAT, Quantitative Scale for Grading of Cardiovascular Autonomic Reflex Tests and Small Fibers from Skin Biopsies; ENFD, Epidermal nerve fiber density; SGNFD, Sweat gland nerve fiber density; SFN, Small fiber neuropathy. %, percent of abnormal results. ^a^Calculated using chi-squared test. ^b^Calculated using Fisher Exact test. Data are mean ± sd; % = percent of abnormal findings.

### 3.3 Invasive cardiopulmonary exercise testing (iCPET)

iCPET was done in a sitting position and results were available in 19 HYCH and 16 POTS patients (Table 7). Stroke volume, cardiac output, oxygen uptake, and right atrial pressure were similar between HYCH and POTS at sitting rest and during exercise. The prevalence of preload failure (100% in both groups), deconditioning (57.9 versus 62.5%, *p* = 0.99), and impaired peripheral oxygen extraction (suggesting peripheral left to right shunting and/or mitochondrial dysfunction) were equal in HYCH and POTS. POTS had elevated heart rate (*p* = 0.004) during sitting rest, but the exercise heart rate was similar to HYCH. Supine heart rate obtained from the autonomic testing showed no difference between HYCH and POTS in these patients who completed ICPET.

**Table 7:** Invasive cardiopulmonary exercise testing in HYCH and POTS.

Variable	HYCH (*n* = 19)	POTS (*n* = 16)	*p*-value^a^
Rest stroke volume, mL	73.81 (25.64)	76.42 (17.30)	0.731
Exercise stroke volume, mL	75.28 (17.48)	67.56 (13.06)	0.155
Difference in stroke volume (exercise-rest), mL	1.48 (28.49)	−8.86 (16.99)	0.212
Supine heart rate from autonomic testing, bpm	76.42 (15.85)	73.69 (14.79)	0.604
Rest heart rate, bpm	81.37 (8.92)	92.44 (10.55)	0.002
Exercise heart rate, bpm	160.11 (18.12)	165.69 (17.17)	0.359
Rest cardiac output, l/min	5.93 (1.94)	6.98 (1.49)	0.085
Exercise cardiac output, l/min	12.16 (3.56)	11.23 (2.41)	0.381
Rest VO_2_, mL/min	278.05 (62.63)	320.50 (53.61)	0.041
Exercise VO_2_, mL/min	1423.37 (630.41)	1349.12 (461.33)	0.698
Rest right atrial pressure, mmHg	−0.84 (1.64)	−0.50 (1.59)	0.538
Exercise right atrial pressure, mmHg	0.53 (2.82)	0.50 (2.34)	0.976
Preload failure, % abnormal	100.0	100.0	0.977
Peak VO_2_, % predicted	78.37 (27.20)	78.12 (20.06)	0.572
Deconditioning, % abnormal	63.2	62.5	0.487
Peak cardiac output, % predicted	98.54 (26.27)	94.14 (17.37)	0.686
Anaerobic threshold, % predicted	46.63 (15.09)	43.19 (13.57)	0.957
Peripheral oxygen extraction	0.85 (0.15)	0.87 (0.12)	0.731
Mitochondrial myopathy, % abnormal	31.6	25.0	0.155

VO_2_, oxygen uptake. Deconditioning was defined as the predicted peak oxygen uptake < 85%, preload failure was defined as right atrial pressure < 6.5 mmHg, mitochondrial myopathy was defined as (CaO_2_ – CvO_2_)/Hb < 0.8, where CaO_2_, arterial oxygen content, VaO_2_, venous oxygen content, Hb, hemoglobin. ^a^Calculated using ANOVA or Fisher exact test as appropriate. Data are mean±sd, %=percent of abnormal findings.

## 4 Discussion

Orthostatic intolerance without orthostatic hypotension is common but only about 50% of patients are diagnosed with POTS on autonomic testing using conventional blood pressure and heart rate monitoring (4, 24, 25). By expanding autonomic testing with cerebral blood flow and end-tidal CO_2_ monitoring, as defined in the Brigham protocol, many patients with orthostatic intolerance without orthostatic tachycardia, satisfy diagnosis of HYCH (4). In this study, we refined our previous findings describing the main features of HYCH (4). The main finding is the presence of multiple similarities between HYCH and POTS thus supporting the concept that POTS and HYCH represent a spectrum of the same disorder.

This study showed typical POTS findings (26) including a predominance of younger women with reduced orthostatic CBFv and end-tidal CO_2_, limited but widespread dysautonomia, and evidence of small fiber neuropathy. Comparing HYCH with POTS, demographics, comorbidities, and medications were similar.

Younger females represent the majority of HYCH patients in both groups and this prevalence has been shown in other POTS studies (6). HYCH patients were treated using similar medication as POTS patients even before the autonomic testing defined diagnoses. The common medications were anti-tachycardic (adrenergic beta-blockers, calcium channel blockers, or ivabradine) as well as medication that increases orthostatic blood pressure (pyridostigmine, proamatine or fludrocortisone). Patient-reported surveys showed similar frequency and intensity of autonomic and sensory symptoms.

Our study also showed that central sensitization syndrome (27) affects the majority of HYCH and POTS patients. Central sensitization increases responsiveness of the nervous system, it may amplify autonomic symptoms and can be an explanation for the mismatch between subjective and objective dysautonomia observed in HYCH, POTS, and other autonomic disorders (28).

Key objective cerebrovascular, capnographic, autonomic, and skin biopsy features were also similar in HYCH and POTS. Both syndromes have a comparable orthostatic decline in CBFv – without orthostatic hypotension. Both groups hyperventilated during the tilt test as evidenced by reduced orthostatic end-tidal CO_2_. POTS patients hyperventilated more often when compared in percent (10.4% versus 12%) but on absolute scale the orthostatic decline of end-tidal CO_2_ was similar (11.98 versus 12.95 mmHg). The relative difference can be explained by difference in baseline end-tidal CO_2_, as POTS patients were slightly hyperventilating during baseline supine position.

The distribution and severity of dysautonomia were also similar. Both groups had widespread but mild dysautonomia affecting all three measured domains: cardiovagal, adrenergic, and sudomotor. Skin biopsy showed similar frequency and severity of neuropathic changes. Finally, norepinephrine and inflammatory markers were also similar between both conditions. Elevated norepinephrine was reported in POTS (29) and may reflect compensatory adrenergic activation to decreased venous return to the heart (30). Although we did not obtain norepinephrine in controls, our study shows a similar level of norepinephrine in HYCH compared to POTS, indicating a similar level of cardiovascular adrenergic tone (31).

This study provides an additional line of evidence that symptomatic cerebral hypoperfusion is a unifying feature of HYCH and POTS. Orthostatic lightheadedness/dizziness is a major sign of cerebral hypoperfusion (3, 32). In this study, orthostatic lightheadedness/dizziness was accompanied by reduced CBFv in both HYCH and POTS during the head-up tilt test. Our previous study (33) showed that changes in CBFv (in addition to end-tidal CO_2_ level that modulates the size of cerebral vessels) indeed correlate with symptoms of cerebral hypoperfusion indicating that reduced CBFv leads to cerebral hypoperfusion.

Reduction in orthostatic CBFv by 19% or more from the supine baseline period is associated with symptoms of central nervous system dysfunction in POTS (32, 34). CBFv decline exceeded that threshold in our HYCH and POTS patients. The mechanism of reduced orthostatic CBFv was also similar across both conditions and is due to hypocapnia-induced cerebral arteriolar vasoconstriction. In a healthy CO_2_ cerebrovascular reactivity, a decrease of end-tidal CO_2_ by 1 mmHg decreases cerebral blood flow by about 3% (35). Our HYCH/POTS subjects had a maximal decrease in orthostatic end-tidal CO_2_ around 12/13 mmHg with a predicted decrease in CBFv by 36/39%, which is close to what was observed in our study (HYCH/POTS decrease by 33/37%). In addition to the reduction of cerebral blood flow and resulting hypoperfusion, respiratory alkalosis associated with hypocapnia may affect brain activity (35, 36). Brain hypoperfusion and related ischemia may drive a central sensitization syndrome that we identified in majority of HYCH and POTS patients.

iPET is an invasive test that monitors ventilation, pulmonary and systemic gas exchange, and hemodynamics during upright incremental exercise (23). iCPET enables to evaluation of peripheral tissue oxygen extraction and to detection of a lower venous return to the right heart termed preload failure. Previous studies showed that most POTS patients exhibit preload failure, a low stroke volume, reduced left ventricular mass, and reduced peak oxygen uptake when upright or exercising (22, 37, 38). Although our iCPET sample size was limited, it showed important similarities between HYCH and POTS in key variables including stroke volume, preload failure and deconditioning.

Preload failure can be due to increased venous capacitance/venous pooling associated with small fiber neuropathy, and hypovolemia, and both factors can be present in POTS (26). Our study confirmed preload failure in POTS but also showed preload failure in HYCH. Considering that both HYCH and POTS have frequent SFN (depending on the definition, the prevalence of SFN is equal or higher in HYCH compared to POTS), our finding is consistent with increased venous capacitance as a cause of preload failure in HYCH and POTS. An alternative explanation of preload failure is dehydration/hypovolemia. Since we did not measure the hydration status in our patients directly, the additional effect of hypovolemia on preload failure cannot be ruled out.

If HYCH and POTS share the same pathophysiology, then why is orthostatic tachycardia present in POTS and not in HYCH?

First, orthostatic tachycardia as defined for the POTS criteria (30 bpm for age > 19 years) is not necessarily an abnormal finding as it can be also seen in healthy subjects (9, 39). Second, it is important to realize that both HYCH and POTS groups have similar abnormalities in peripheral domains including SFN, intensity, and distribution of dysautonomia, and other key variables that affect the heart rate such as stroke volume, peak oxygen uptake at exercise, the prevalence of deconditioning, and peripheral oxygen extraction. It was proposed that the orthostatic tachycardia in POTS compensates for the low stroke volume to maintain cardiac output (22) since cardiac output is defined as stroke volume multiplied by heart rate. This concept cannot explain the lack of orthostatic tachycardia in HYCH because the stroke volume, neuropathic, and cardiovascular peripheral variables influencing heart rate are equal in HYCH and POTS. Therefore, we infer that orthostatic tachycardia in POTS results from the central nervous system/brain driven overcompensation of the orthostatic challenge. The iCPET showed increased resting heart rate in POTS in a sitting position, but supine heart rate and stroke volume were equal in HYCH and POTS. Since the sitting position is semi orthostatic the finding of increased sitting heart rate in POTS during iCPET is consistent with a centrally driven tachycardic overcompensation.

The concept of the overcompensatory orthostatic tachycardia in POTS is consistent with a clinical experience as POTS patients in general benefit from antitachycardic medication (1).

Opposite scenario, e.g., wheatear the tachycardia in POTS is an adequate compensatory response, and the lack of tachycardia in HYCH is a sign of undercompensation, is unlikely for the following reasons: (1) there was no evidence of chronotropic incompetence, e.g., inability to increase the heart rate as needed during exercise (40), as HYCH patients were able to increase the heart rate during exercise to similar level as POTS; (2) HYCH patients had normal increase of the orthostatic heart rate comparable to healthy controls during the tilt test; (3) Portion of HYCH patients were on antitachycardic mediation, which should worsen the HYCH if the lack of tachycardia is undercompesation.

### 4.1 Differences between HYCH and POTS except of orthostatic tachycardia

Although key hemodynamic and neuropathic variables were similar in HYCH and POTS, there were some differences in gastrointestinal and respiratory systems.

#### 4.1.1 Gastrointestinal system

HYCH had more frequent irritable bowel syndrome than POTS patients (although the difference did not reach statistical significance) and HYCH patients were using more frequently gastrointestinal medication. HYCH also had more frequent sudomotor abnormalities. The above results imply differential involvement of the gastrointestinal system in HYCH. Nevertheless, the key variables that are related to gastrointestinal system which uses cholinergic transmission, such as sweat gland nerve fiber density and severity (not frequency) of sudomotor abnormalities were similar in HYCH and POTS. The difference in gastrointestinal domain may reflect a referral bias, this question could be evaluated in feature studies.

#### 4.1.2 Hyperventilation

POTS patients were slightly more hyperventilating during baseline supine position, and were also hyperventilating more on relative scale and longer (as indicated by QASAT grading) during the tilt. Although it was reported that hyperventilation is the cause of orthostatic tachycardia in POTS (41), our study suggest that hyperventilation can be a contributing factor but cannot explain the tachycardia as a sole cause. Only POTS patients were hyperventilating in supine position, yet the POTS and HYCH patients had similar supine heart rate. Orthostatic decline in end-tidal CO_2_ was similar in both groups on absolute scale, but only POTS had elevated orthostatic heart rate. However, hypocapnia has important effect on the CBFv as discussed above.

#### 4.1.3 The role of cerebral blood flow in orthostatic intolerance

Our study provides additional evidence of importance of cerebral blood flow and respiratory system in orthostatic intolerance syndromes without orthostatic hypotension. CBFv monitoring and capnography can increase our understanding of common orthostatic syndromes where conventional heart rate and blood pressure monitoring has limited value.

### 4.2 Study limitations

A referral bias and retrospective character of the study may affect the selection of subjects; therefore, the results may not represent the whole POTS and HYCH population. However, our sample is representative of POTS population given the similarities with other studies.

We did not measure blood volume directly and central hypovolemia can results in orthostatic tachycardia (41). However, the iCPET showed similar right atrial pressure and similar frequency of preload failure in both conditions. This suggest that HYCH and POTS had similar blood volume since both right atrial pressure and preload failure is affected by blood volume (42). Cerebral blood flow was assessed indirectly using Transcranial Doppler which measures flow velocity and not flow. The velocity is proportional to blood flow assuming that the diameter of the insonated vessel does not change during orthostatic stress, which was confirmed by an imaging study (43). CBFv is also affected by the angle of the TCD probe. Although the angle varies from patient to patient, the same angle was used throughout the testing once the probe was properly positioned.

## 5 Conclusion

Our findings support the hypothesis that HYCH and POTS represent a spectrum of the same disorder of cerebral hypoperfusion. Reduced cerebral blood flow due to venous pooling and related hypocapnia may be a physiological substrate. Orthostatic tachycardia in POTS is likely driven by the central nervous system’s overcompensation of orthostatic challenge. Cerebral blood flow monitoring is necessary to identify and characterize orthostatic intolerance disorders.

## Data Availability

The raw data supporting the conclusions of this article will be made available by the authors, without undue reservation.
